# Supplementary material for: Mechanisms governing the pioneering and redistribution capabilities of the non-classical pioneer PU.1
Source: Nat Commun. 2020 Jan 21;11:402. doi: 10.1038/s41467-019-13960-2 (PMC6972792; doi:10.1038/s41467-019-13960-2)
Supplement: Supplementary file 7 — Source data [file 41467_2019_13960_MOESM7_ESM.zip › Source_Data/Figure6/Figure6B_MotifScanOutput/ETS-specific/homerResults/motif13.similar.html]

motif13

## Information for motif13

C
A
G
T
T
A
C
G
G
A
C
T
C
T
A
G
C
A
T
G
T
C
A
G
C
T
A
G
C
T
A
G
G
T
C
A
C
T
A
G
A
C
T
G
T
A
G
C
  
Reverse Opposite:  

A
T
C
G
T
G
A
C
G
A
T
C
C
A
G
T
A
G
T
C
G
A
T
C
A
G
T
C
G
T
A
C
A
G
T
C
C
T
G
A
A
T
G
C
G
T
C
A
  

|  |  |
| --- | --- |
| p-value: | 1e-88 |
| log p-value: | -2.049e+02 |
| Information Content per bp: | 1.611 |
| Number of Target Sequences with motif | 1973.0 |
| Percentage of Target Sequences with motif | 25.77% |
| Number of Background Sequences with motif | 6407.8 |
| Percentage of Background Sequences with motif | 15.88% |
| Average Position of motif in Targets | 101.7 +/- 53.5bp |
| Average Position of motif in Background | 99.7 +/- 60.2bp |
| Strand Bias (log2 ratio + to - strand density) | -0.1 |
| Multiplicity (# of sites on avg that occur together) | 1.22 |
| Motif File: | file (matrix) reverse opposite |

### Similar de novo motifs found

|  |  |  |  |  |  |  |  |
| --- | --- | --- | --- | --- | --- | --- | --- |
| Rank | Match Score | Redundant Motif | P-value | log P-value | % of Targets | % of Background | Motif file |
| 1 | 0.964 | C A G T T A C G G A C T A C T G C A T G C A T G T C A G C T A G T G C A C T A G A C T G | 1e-88 | -203.245536 | 23.62% | 14.14% | motif file (matrix) |
| 2 | 0.754 | C T A G C A T G T A C G T A C G T C A G T C A G C T A G T C A G | 1e-87 | -200.364944 | 52.25% | 39.98% | motif file (matrix) |
| 3 | 0.953 | A G C T T A C G A G C T A C T G C A T G T A C G C T A G A C T G G C T A C A T G | 1e-80 | -184.827075 | 16.29% | 8.74% | motif file (matrix) |
| 4 | 0.767 | A T G C G A T C A G T C A T G C A G T C T A G C G T A C G A T C G C A T | 1e-75 | -174.876016 | 24.17% | 15.21% | motif file (matrix) |
| 5 | 0.782 | A C T G A C T G A T C G T A C G T C A G C T A G A C T G | 1e-75 | -172.894855 | 36.96% | 26.42% | motif file (matrix) |
| 6 | 0.948 | T A C G G A C T A C T G C A T G T A C G A C T G A C T G C G T A C T A G | 1e-72 | -166.572675 | 14.63% | 7.81% | motif file (matrix) |
| 7 | 0.846 | G T C A A G T C A C G T G T A C A G T C A G T C G T C A A G T C | 1e-47 | -110.441371 | 11.03% | 6.11% | motif file (matrix) |
| 8 | 0.812 | A G T C A G C T A G T C A G T C A G T C C G T A A G T C | 1e-35 | -82.016525 | 19.88% | 14.11% | motif file (matrix) |
| 9 | 0.924 | A T G C G C A T A T C G G A C T A C T G A C T G C T A G C T A G T A C G C G T A C T A G T A C G T A G C | 1e-28 | -66.299451 | 1.54% | 0.35% | motif file (matrix) |
| 10 | 0.768 | C T G A T A C G C A T G A C T G C T A G A C T G G C T A A C T G A C T G C T G A A C T G C T A G | 1e-27 | -64.468632 | 6.98% | 3.96% | motif file (matrix) |
